# Nutritional Strategies to Alleviate Stress and Improve Welfare in Dairy Ruminants

**DOI:** 10.3390/ani14172573

**Published:** 2024-09-04

**Authors:** Basiliki Kotsampasi, Maria Anastasia Karatzia, Dimitrios Tsiokos, Stella Chadio

**Affiliations:** 1 Research Institute of Animal Science, Directorate General of Agricultural Research, Hellenic Agricultural Organization—DIMITRA, 58100 Giannitsa, Greece; karatzia@elgo.gr (M.A.K.); tsiokosd@elgo.gr (D.T.); 2 Laboratory of Anatomy and Physiology of Farm Animals, Department of Animal Science, School of Animal Biosciences, Agricultural University of Athens, Iera Odos 75, 11855 Athens, Greece; shad@aua.gr

**Keywords:** dairy ruminants, transition period, heat stress, welfare, fatty acids, amino acids, feed additives, nutraceuticals

## Abstract

**Simple Summary:**

The intensification of the dairy sector, along with climate change and the rising ambient temperature, has exposed dairy ruminants to a variety of stress factors that they must cope with to maintain optimum physiology, performance, health, and well-being. The use of functional fatty acids, amino acids, and feed additives (minerals, prebiotics, probiotics, essential oils and herbs, phytobiotics, enzymes, etc.) on ruminants’ diets has been found to be helpful in overcoming the negative effects of stress that animals are exposed to during critical periods, like transition periods and heat stress. The dietary inclusion of these components, in addition to other management practices, would help livestock farmers overcome the stress challenges ruminants are faced. These components have been considered to regulate and enhance the compromised immunological, metabolic, and oxidative status that occurs in animals exposed to metabolic disturbances and heat-stressed environments. Thus, ruminants’ supplementation with the aforementioned nutrients is regarded as a key nutritional strategy for alleviating stress and ensuring optimum milk production and reproduction efficiency, as well as health and welfare. Succeeding this, dairy farm profitability and sustainability will be guaranteed, and the public demand for animal wellbeing and welfare will be met.

**Abstract:**

Dairy ruminants provide a major part of the livestock and agriculture sectors. Due to the increase in world population and the subsequent increase in dairy product demands, the dairy sector has been intensified. Dairy farming intensification and the subsequent increase in animal nutritional demands and the increase in the average global temperature as well have subjected animals to various stress conditions that impact their health and welfare. Various management practices and nutritional strategies have been proposed and studied to alleviate these impacts, especially under heat stress, as well as during critical periods, like the transition period. Some of the nutritional interventions to cope with stress factors and ensure optimal health and production are the inclusion of functional fatty acids and amino acids and feed additives (minerals, prebiotics, probiotics, essential oils and herbs, phytobiotics, enzymes, etc.) that have been proven to regulate animals’ metabolism and improve their antioxidant status and immune function. Thus, these nutritional strategies could be the key to ensuring optimum growth, milk production, and reproduction efficiency. This review summarizes and highlights key nutritional approaches to support the remarkable metabolic adaptations ruminants are facing during the transition period and to reduce heat stress effects and evaluate their beneficial effects on animal physiology, performance, health, as well as welfare.

## 1. Introduction

Ruminants are a vital part of the world’s food security and nutrition, as well as the livelihoods of farmers and others involved in the agrifood chain. The majority of the world’s livestock production is comprised of ruminant species, such as cattle, sheep, goats, and buffalo, that provide more than half of the protein obtained from the livestock industry, primarily in the form of milk and meat [1]. From 1961 to 2022, world raw cattle milk production has increased from 313 to 753 million tons, that of goats from 6.97 million to 19.19 million tons, of sheep from 5.10 to 10.09 million tons, and of buffaloes from 17.86 to 143.57 million tons [2]. The significant rise in milk production can be attributed to the continuous selection toward higher milk yield along with improvements in management, housing, feeding, and veterinary care [3,4]. However, the increase in milk yield has been accompanied by declining fertility, increasing incidence of mastitis, leg, and metabolic diseases, and declining longevity, thus modifying normal behavior, indicative of a substantial decline in animal welfare [5]. Indeed, it has been proven that maximization in milk yield leads to oxidative stress and immunosuppression in ruminants [5], affects animals’ welfare, increases animals’ disease susceptibility, and reduces animals’ fertility and ability to reach their peak milk yield, thus negatively impacting dairy farms [6,7]. Therefore, high-yielding dairy ruminants are exposed to various stress-induced factors that must be tackled in order to secure animal productivity, health, and welfare. Improving welfare is important, as good welfare is regarded by the public as indicative of sustainable systems and good product quality and may also be economically beneficial [5].

Concurrently, there is an increasing public and scientific concern on the nonessential use of antibiotics in livestock farming and the possible increased antibiotic resistance towards both animals and humans’ pathogens. The administration of antibiotics in ruminant breeding is a common routine to mitigate the negative effects of animal deceases, such as increased mortality, reduced productivity, or spreading to other animals [8]. Besides the therapeutical use, antibiotics are also used as prophylactic measures to prevent infection in animals at risk during stressful situations and as a growth promoter to improve feed efficiency and productivity [9], although this practice has been banned in the European Union (EU, 1831/2003) and several other developed countries [10]. The overuse of antibiotics in food animals has led to the development of bacterial resistance and the widespread of resistant bacteria, which are a serious threat to animal and public health [11]. Restrictions on use of antibiotics in food animal production and the promotion of responsible use have been issued in several international and national guidelines in order to ensure the quality of treatments but also to mitigate resistance [12]. Alongside policies, the implementation of preventive measures while adopting farming practices that promote animal welfare is of utmost importance to reduce the use of antibiotics through disease avoidance. In recent years, research for natural products and feed additives, such as phytobiotics, probiotics, prebiotics, etc., to substitute antibiotic use for disease prevention and production enhancement is gaining significant prominence [13].

Furthermore, the growing demand for healthy animal products along with increasing awareness in sustainable production highlights the need for optimizing animal nutrition. To this respect, among the different developing solutions, feed additives that affect animal productivity, welfare, and health are gaining significant importance [14].

### 1.1. Relation of Stress and Animal Welfare

According to the Terrestrial Code of the World Organization for Animal Health (WOAH), animal welfare refers to an animal’s physical and mental state in relation to the conditions in which it lives and dies. It has been described in a variety of ways, but one succinct definition that covers all bases is that it is the state in which animals are healthy and have all their needs addressed. Animal welfare has been established as an important public awareness subject, leading to a demand for high welfare standards by all relevant professionals and consumers alike [15].

Welfare is a quantifiable characteristic of the state of the animal at any given point, which varies from poor to very good and can be defined by discrete measures, such as changes in hormone concentration, body temperature, and normal behavior. Poor welfare will develop when an individual has difficulty coping with its environment. There is a lot of overlap between productive and welfare measures, such as disease, mortality risk, growth, milk yield, and reproduction [16].

Sustainable livestock production methods must incorporate animal welfare, especially considering modern consumer concerns about products produced under unacceptable conditions. In any effort to increase production efficiency, animal welfare must be given high consideration along with other sustainability strategies [15]. Therefore, it is crucial to adopt strategies that not only advance sustainable productivity but also improve animal welfare. Such strategies include feed supplementation and animal health enhancement [17]. Especially, nutrition has been acknowledged as the most fundamental requirements for an animal’s wellbeing, regardless of breed, productivity level, health, or farm.

### 1.2. Stress Factors in Ruminants

All farm animals will experience some level of stress during their lives. The term stress implies the system of biological reactions in mammals based on the interaction of two factors: the adverse effect of a stimulus on one side and the protective response and reactions to the other. Stress is an organism’s non-specific way of adapting to the negative impacts of a variety of internal and external factors. It is a complicated reaction that protects most life processes and functions within their normal range. Stress, however, has the potential to seriously impair the body’s overall systems and organ functions. There are clear functional abnormalities and behavioral alterations to the physiological changes brought on by the stressor. Through a variety of hormones, neurotransmitters, and neuropeptides, the hypothalamic–pituitary–adrenal axis (HPA), the autonomic nervous system, and, thereby, the sympathetic–adrenal–medullary (SAM) axis regulate the stress response by controlling the physiological process according to a predetermined time course and specificity for each stressor that triggers the reaction [18,19]. Naturally, farm animals will be challenged by different stressors and, consequently, develop varying degrees of stress responses during their lives. Although there is no standard classification system for stressors, they can be categorized into two categories based on their source: internal stressors, which originate from the animal’s body, and external stressors, which originate from the animal's environment. Some of the common factors that produce stress when they act within any animal production system include inadequate nutrition, deprivation of water and/or feed, heat, cold, overcrowding, and handling (i.e., interaction with humans or human manipulation of the animals) [19]. These factors, if persistent and exacerbate animals’ homeostasis, could lead to oxidative stress. Under normal physiological conditions, oxidation processes provide energy and help in cellular defense actions that are necessary for the maintenance of natural life by producing components that are derived from oxygen, collectively termed as ‘free radicals.’ Reactive oxygen species (ROS) and reactive nitrogen species (RNS), derived from oxygen and nitrogen, respectively, are the most common groups of free radicals in biological systems. The positive effects of free radicals occur when they are produced at low or moderate concentrations within biological systems, as they are involved in many physiological roles, such as in cellular responses and maintenance. However, a stimulus that results in their excess can lead to non-controlled oxidation in the cells and tissues of biological systems, known as oxidative stress. In such circumstances, these radicals can damage biological macromolecules and form primary and secondary compounds that can multiply the formation of radicals and are destructive to the cellular or tissue systems and their metabolic activities [20]. Heat stress and metabolic disorders during the transition period in ruminants have been found to cause oxidative stress, increasing their susceptibility to pathogens and metabolic diseases. Thus, in the current review, nutritional strategies to ameliorate stress conditions during the transition period and heat stress will be provided.

#### 1.2.1. Transition Period in Dairy Cows

The peripartum or transition period in dairy cows, that lasts 3 weeks before to 3 weeks after parturition, is considered a challenging phase during which remarkable metabolic, endocrine, physiologic, and immune adaptations occur to support lactation and avoid metabolic dysfunction [21,22]. Dairy cows at that period are subject to the imbalance between energy demands for the synthesis of colostrum and milk and dietary energy intake due to depression of feed intake and experience negative energy balance (NEB), resulting in stimulation of fat mobilization. This inevitably causes a shift from anabolic to catabolic metabolism. Triglycerides, the main lipid molecules stored as an energy reserve in adipose tissue, undergo a biochemical hydrolysis pathway mediated by lipases. Triglyceride hydrolysis releases glycerol and non-esterified fatty acid (NEFA) molecules, which are the major source of energy for the tissues during periods of NEB [23]. Although these changes are a normal adaptive process in high-yielding cows, when a cow fails to adapt to this metabolic challenge, excessive concentration of NEFA is noticed [23] that leads to oxidative stress [24], rumen acidosis, protein metabolism imbalance, and immunological and inflammatory abnormalities [25,26,27,28]. Together, these factors compromise animal health, productive efficiency, and udder health during periparturient phases, reduce the milk yield and reproductive efficiency of cows, and cause serious economic losses [21,29,30,31,32].

Therefore, a safe transitional period by maintaining redox homeostasis during the periparturient and peak lactation periods is of high importance to secure the efficient subsequent production and reproduction performance of dairy cows. Conversely, if dairy cows fail to adapt to this metabolic challenge, several infectious disorders occur linked to rising rates of health issues and, as a result, financial losses for dairy farmers [33,34,35,36]. The practical goal of nutritional management during the transition period is to support these metabolic adaptations that are essential for successful reproductive performance and fetus development, as well as the provision of the nutrient amounts of energy, protein, and macro/micronutrients that are required during gestation and following parturition [21,22].

Several studies have highlighted the effectiveness of various nutrients and feed additives (including amino acids, essential fatty acids, probiotics and prebiotics, antioxidant vitamins and trace minerals, and phytoactive compounds) at the crucial period of the transition to enhance the metabolic, immune, and antioxidant system response after parturition for the improvement of animal health, welfare, and productivity in herd health management, which will be discussed in the following sections. For example, essential fatty acid supplementation for two months—one month prior to and one month following calving—successfully reduces the inflammatory status of cows. The advantageous effects of methyl donors, such as methionine and choline, have been shown to target the inflammatory and immunological response. These effects both directly and indirectly modulate the response by increasing the levels of antioxidants glutathione (GSH) and taurine. Nutraceuticals have been shown to control the immunological response, thereby alleviating the detrimental consequences of parturition stress and the problems that follow, which will be discussed in the following sections [22].

#### 1.2.2. Heat Stress

Global climate change, which is characterized by a constantly increasing environmental temperature [37], has a negative impact on the livestock sector, affecting animals’ welfare and productivity as it reduces fertility, suppresses the immune system, and increases the susceptibility to infections and diseases [38,39,40]. Animals experience heat stress when the ambient temperature exceeds their thermoneutral zone, compromising their ability to dissipate metabolically produced heat and maintain thermal balance [41]. High ambient temperature in combination with high relative humidity, expressed as temperature humidity index (THI) [42], has been ordinarily used as an indicator for the degree of heat stress [43], although a number of other environmental factors, such as solar radiation and wind speed, contribute to heat stress as well [44].

Vulnerability to heat stress varies according to species, with large ruminants, particularly dairy cows, being more susceptible than small ruminants [45], mainly due to their high metabolic rate associated with milk synthesis. However, although sheep and goats can adapt to the heat stress more easily, their productivity is often compromised [46]. The effects of heat stress on livestock production and reproduction have been extensively investigated, and a number of recent reviews summarize the existing evidence [40,47].

Animals respond to heat stress by activating a variety of physiological, morphological, behavioral, and productive and reproductive responses [48]. Metabolic adaptations to combat heat stress lead to the activation of the inflammatory immune system and oxidative stress [49], and therefore the development of specific strategies to ameliorate the negative effects is of significant importance. Among the different approaches, nutrition interventions, especially the provision of a well-balanced fiber to concentrate ratio in the diet, along with optimum protein and energy content, are crucial, as heat stress is well known to reduce feed intake and energy availability [50,51,52]. In addition, during the last years, different nutrients and feed additives, such as amino acids, antioxidants, minerals, vitamins, and plant extracts, have been tested for their ability to eliminate the negative effects of heat stress [53,54]. As an example, the provision of specific amino acids, like glutamine and glutamate, seems to exert beneficial effects, as they are important for immune response, particularly during sensitive periods, such as the transition period in dairy animals [55].

Antioxidant supplementation has also been reported to be beneficial in terms of reproductive efficiency [56,57], as heat stress has a negative impact on reproductive success by compromising the quality and competence of the oocyte, reducing fertility, and impairing embryonic development [47,58,59]. In the long term, genetic interventions provide a chance to identify reliable biomarkers for heat stress resilience, which could be incorporated into selection programs for thermotolerance [60].

## 2. Nutritional Strategies to Alleviate Stress

### 2.1. Functional Amino Acids

Amino acids (AAs) are the building blocks of proteins and other nitrogenous substances, glucose, and fatty acids required by ruminants to ensure optimum growth, reproduction, lactation, and maintenance [61]. However, apart from this role, specific AAs act as activators of cell signaling that influence key metabolic pathways and important physiological functions [62]. Methionine and Lysine are generally considered the first limiting AA for production in ruminants, and their protected form has been widely studied for their role in the maintenance of animals’ antioxidant and immunity status and anti-inflammatory ability, thus associated with better health and productive efficiency and welfare maintenance [62,63,64,65,66,67,68]. They act as methyl donors for the production of the most important extracellular source of the antioxidants GSH and taurine [69], essential for maintaining the cellular redox homeostasis, meaning the essential and dynamic process that ensures the balance between reducing and oxidizing reactions within cells [70,71,72,73]. Furthermore, rumen-protected limiting amino acids are necessary for the biosynthesis of S-adenosylmethionine (SAM) [74], which is required for many biological processes, such as transsulfuration, polyamine biosynthesis, and DNA methylation [75]. Therefore, enhanced SAM production due to amino acid supplementation can help enhance the flux of the transsulfuration pathway, increasing taurine and GSH production to help reduce oxidative stress as part of 1-carbon metabolism pathways. Another key role of amino acids is regarded as the enhancement of immune function, as they play an important role in immunological responses by regulating the activation of T lymphocytes, B lymphocytes, natural killer cells, and macrophages, cellular redox state, gene expression, lymphocyte proliferation, and the production of antibodies, cytokines, and other cytotoxic substances [69].

Choline, also called trimethyl ethanolamine, is a nutrient with an amino acid-like structure and metabolism. It is regarded as an essential nutrient for ruminants since it is required to produce acetylcholine, a neurotransmitter, and SAM. Choline is a donor of betaine, which in turn provides a methyl group for the conversion of homocysteine to provide methionine. Choline shares the same biological functions with methionine and lysine [76].

#### 2.1.1. Amino Acid Supplementation during Transition Period in Ruminants

Various studies have examined the potential of amino acid supplementation as a feeding strategy to enhance the metabolic, immune, and antioxidant system responses of dairy cows during the transitional period. Rumen-protected amino acid supplementation periparturient has been associated with positive health responses given that they represent immunomodulatory properties. Indeed, rumen protected Methionine (RPM) supplementation of dairy cows periparturient, 21 days before the expected calving to 30 days in milk, in the form of MetaSmart (MS) or Smartamine M (SM) at a rate of 0.19% and 0.07% of dry matter (DM), respectively [77,78,79,80], or with ethyl-cellulose RPM from −28 to 60 day relative to parturition with 0.09% and 0.10% of DM, during the prepartum and postpartum period, respectively [81] and from −21 to 21 day relative to parturition with 15 g/day RPM [82], has been found to improve the concentrations of plasma biomarkers of inflammation, such as decrease in interleukin-1b (IL-1β), interleukin-6 and haptoglobin concentration, increase in albumin concentration and in neutrophil phagocytosis capacity and increase in proliferative ability of peripheral blood T lymphocytes, enhance of immunometabolic status and reduce proinflammatory signaling within liver. Moreover, alleviated hyper response of inflammatory cytokines was observed around parturition due to oxidative stress in dairy cows supplemented with RPM (at a rate of 0.08% of DM) 21 days before to 30 days relative to parturition [83], indicating that the increased dry matter intake and milk yield observed after RPM supplementation could be due to the reduction of inflammation and oxidative stress. RPM along with Choline (RPC) supplementation, 21 days before to 21 days after parturition, at inclusion levels of 15 g/day RPM and 15 g/day RPC enhanced immune function of transition dairy cows as indicated by the elevated plasma interleukin 2 (IL-2) concentration and the CD4+/CD8+ T lymphocyte ratio and the decrease in the levels of tumor necrosis factor-α (TNF-α) and IL-6 [82], whereas dairy cows supplementation with 4 g of digestible RPM and 10 g of digestible rumen protected lysine (RPL) per cow per day, three weeks before to three weeks after parturition, has been reported to reduce milk somatic cell count and improve immunity and health status of animals [84].

Further, previous studies have proved the enhancement of the antioxidant status of transitional cows after AA supplementation. RPM supply of cows during the periparturient period resulted in an increase in plasma concentrations of ferric-reducing antioxidant power, β-carotene, tocopherol, and total and reduced GSH [81], while reactive oxygen metabolites (ROM) decreased [79,83]. Sun et al. (2016) [82] also observed an improvement in blood antioxidant status, with RPM increasing total antioxidant capacity, glutathione peroxidase (GPX) activity, and vitamin E. Dairy cows supplementation from day −28 to 60 relative to parturition with RPM at a rate of 0.09% and 0.10% of DMI during the prepartum and postpartum periods has also been proved to have positive effects on mammary glad antioxidant status, as indicated by the greater mRNA abundance of specific key target genes that regulate tissue’s antioxidant mechanisms [69] and liver function, which denotes an increase in liver function biomarkers, which are linked to the reduction in inflammation and oxidative stress [78,81]. RPM alone (2.5 g/kg concentrate) or in combination with RPC and rumen protected Betaine (5 g/kg of a commercial product), alleviates oxidative stress by enhancing glutathione transferase (GST) activity in the plasma of periparturient ewes [85] or GSH activity of peripartum dairy cows (−21 to +30 day relative to parturition) when supplemented with RPM adjusted daily at 0.08% of DM of diet and RPC fed at 60 g/cow per day [86].

Furthermore, various studies have demonstrated the antioxidant, anti-inflammatory, and immune-regulatory role of RPC supplementation alone, thus alleviating metabolic stress in dairy cattle during the periparturient period. According to these studies, RPC supplementation (providing 14.4 g/day, or 12.9 g/day, or 25 to 50 g/day) of dairy cows during the transition period (3 wk before to 3 or 6 or 8 weeks after calving) has been found to improve liver biomarkers and metabolic stress, reduce NEFA and β-hydroxybutyric acid (*βHBA*) concentration, resulting in enhanced antioxidative state and maintenance of the anti-inflammatory state of animals, and improved DMI, milk production efficiency, and fertility performance, while maintaining body condition score [87,88,89,90,91,92,93,94,95,96,97].

RPM supplementation of Murrah buffaloes at 10 g/day and 20 g/day/animal, from day 30 prepartum till day 60, resulted in higher conception rates (50.00% (10 g/day/animal), 83.33% (20 g/day/animal), and 33.33% (no supplemented). Varying progesterone and estradiol concentrations were indicative of the positive role of dietary RPM supplementation. According to the authors, dietary RPM improved follicular structures and increased production and reproduction outcomes [98]. RPM alone (10 gm/animal/day) or along with RPC (RPM; 10 gm/animal/day plus RPC; 50 gm/animal/day) supplementation of Surti buffaloes from −15 d to 30 d relative to parturition resulted in increased blood GSH, SOD, and total antioxidant status (TAS), higher in the combined group, and lower blood lipid peroxidation (LPO). Moreover, tumor necrosis factor-α (TNF-α) and haptaglobin during the postpartum period were significantly (*p* < 0.05) lowered in both supplemented groups relative to unsupplemented, suggesting that supplementation of methionine and choline during the transition period might have reduced the inflammatory response. According to the authors, rumen-protected methionine and choline during the transition phase of Surti buffaloes reduces oxidative stress as well as inflammatory tendencies and increases antioxidant status as well as immune response. Beneficial effects of supplementing both were more pronounced than supplementing rumen-protected methionine alone [99].

RPL (pre-calving at 0.33% of DM and post-calving at 0.24% of DM), RPM (pre-calving at 0.16% of DM and post-calving at 0.12% of DM), or the combination RPML (pre-calving at 0.16% RPM + 0.33% RPL of DM and post-calving at 0.12% RPM + 0.24% RPL of DM) throughout the transition period (3 weeks before till 3 weeks after calving), improved DMI, milk yield (41.1 kg/d in RPAA vs. 35.2 kg/d no supplemented) and milk production efficiency, energy-corrected milk and milk chemical composition [100] and had a greater peak of milk yield (52.9, 50.5, and 56.20 kg/day, respectively, in supplemented cows vs. 46.4 kg/day in unsupplemented), reached earlier the peak of milk (55.6, 57.1, and 47.2 days, respectively, in supplemented cows vs. 63.1 days in unsupplemented), and presented higher fertility efficiency as measured by pregnancy rate (22.00%, 22.23%, and 23.74%, respectively, in supplemented cows vs. 20.22% in unsupplemented peripartum) [101].

Although glutamine (Gln) is a non-essential amino acid, its important role in the modulation of immune responses has been well presented in an excellent review by Cruzat et al. (2018) [102]. Gln is used by immune cells to cover their energetic needs, whereas studies have shown that parenteral Gln administration in dairy cows from parturition to seven days postpartum, a period where the animal experiences substantial metabolic stress, was associated with increased concentrations of serum amyloid A (SAA) and lipopolysaccharide-binding protein (LBP) and decreased concentrations of haptoglobin in the plasma of cows, indicating a role for Gln in production of acute phase proteins and maintenance of mucosal barrier functions [103]. Rumen-protected Glutamine (RPGln) supplementation of dairy cows at parturition with 150, 250, and 350 g/day/cow led to greater DMI, milk yield, plasma glucose, total protein, and albumin and better indices of liver function as indicated by the lower aspartate aminotransferase and metabolic stress, as indicated by the lower concentrations of *β*HBA and free fatty acids [104], and improved antioxidant status of the animals as indicated by the higher total antioxidant capacity (TAC) and GSH activity, when cows supplemented 25 days before up to 21 days after parturition with 100 g/day RPGln [105]. Thus, Gln supplementation at the transition period at 100 g up to 350 g/day/cow might be a practical tool for cows to overcome some of the physiological challenges induced during this period.

Table 1 summarizes the effects of amino acid supplementation on various physiological functions of ruminants during the transition period to cope with metabolic and the relative immune and antioxidant disturbances.

Overall, it could be illustrated that rumen protected amino acid supplementation of dairy cows during the transition period could be a practical dietary tool for reducing negative energy balance and improving the metabolic adaptation of peripartum dairy cows, which is crucial for reducing the occurrence of postpartum diseases, enhancing milk yield and reproductive performance, and ensuring animal well-being.

#### 2.1.2. Amino Acid Supplementation to Alleviate Heat Stress in Ruminants

It has been demonstrated that during heat stress, the associated systemic inflammatory response alters the utilization of AAs in tissues, as it increases the synthesis of acute phase proteins and heat shock proteins and promotes their hepatic removal to sustain augmented protein synthesis. Thus, in heat-stressed animals, the increase in intestinal and hepatic AA utilization reduces their availability to peripheral tissues and consequently impairs animals’ production efficiency [118]. In this regard, various studies have demonstrated the beneficial effects of essential and non-essential amino acid supplementation as a strategic tool to alleviate the negative impact of heat stress on ruminants. Dietary supplementation of Lysine, Methionine, and Histidine (179, 58, and 45 g/day, respectively) in dairy cows subjected to heat stress (THI: 82–84) challenged decreased rectal temperature and enhanced intermediary metabolism without any pronounced effects on milk yield and composition [106,107] and decreased leucine oxidation [107], which is considered an essential amino acid for milk production, as is used for protein synthesis [118]. In vitro incubation of heat-stressed bovine mammary epithelial cells with methionine and arginine improved cell functions and mammary metabolism [108], whereas RPM supplementation (1.05 of RPM/kg of DMI) of dairy cows exposed to heat stress challenge with an electric heat blanket (generated a temperature of 36 °C) maintained homeostasis in mammalian targets of rapamycin (mTOR), insulin signaling, and 1-carbon metabolism and whole-blood antioxidant response, important aspects of innate immune function [109]. However, in another study, heat stress-challenged cows supplemented with 1.05 g of RPM/kg of DMI presented a decreased mammary cell apoptosis to proliferation ratio during HSC, but no significant effects were observed on blood immune biomarkers [110]. In dairy goats supplemented with 0, 1.5, 3, 4.5, or 6 g/day RPM and subjected to severe heat stress (THI > 90), enhanced plasma immunoglobulin G concentration, increased milk protein content, and decreased plasma urea–nitrogen concentration at the highest level of supplementation, but only the intermediate levels 1.5 to 4.5 g/day were found to increase feed efficiency ratio and gross profit [111].

Rumen protected Gln supplementation of dairy cows, at 160 g/day, at the summer season, when THI was always higher than 72, enhanced cows’ immune reactions in terms of a strengthening of cell-mediated immune response, which is weakened under heat stress, as indicated by the lower production of IL-10 and the increased in average skinfold after subcutaneous injection with chicken egg albumin [112]. Betaine, a non-toxic amino acid found widely in nature, acts as a methyl donor, participates in protein and lipid metabolism, and is an organic osmoprotectant. Furthermore, betaine is known to have anti-apoptotic properties, thus promoting cell proliferation and preserving gut tissue integrity in heat-stressed cows [113]. Betaine supplementation (2 or 4 g betaine/day) of heat-stressed (cyclical heat exposure to 18–43 °C) sheep resulted in rectal and skin temperatures decreasing and in respiration rates and plasma NEFA concentration reduction, supporting that dietary Betaine supplementation at 2 g betaine/day provides improvements in physiological responses typical of ewes exposed to heat stress and may be a beneficial supplement for the management of sheep during summer [114]. In grazing dairy cows, betaine supplementation (2 g/kg natural betaine in concentrate ration for approximately 3 weeks) improved milk yield and quality during summer [115], while betaine supplementation (15 or 30 g/day per cow) for 60 days in heat-stressed Holstein cows increased feed intake, rumen fermentation, and apparent digestibility and improved the antioxidant profile and serum metabolites, while authors concluded that 15 g of betaine per day can lead to better production performance of dairy cows in heat stress conditions [116].

Betaine supplementation alone (30 g/day) or with bypass fat (30 g and 100 g/day, respectively) of lactating buffaloes under THI ranging from 70.22 ± 0.65 to 80.10 ± 1.07 (maximum 85.24) did not have any effect on milk yield; however, reduction in milk yield was lower in both supplemented groups as compared with control. Milk fat improved by 3.2% and 6.2% in supplemented groups, respectively. Rectal temperature, respiration rate, and pulse rate were significantly lower in buffaloes under heat stress. Average net daily income was increased by 36.02 and 47.43, respectively, as compared with unsupplemented animals [117].

The beneficial effects of amino acid supplementation on ruminants ability to combat heat stress and the physiological responses are summarized in Table 1.

Thus, ruminants could benefit from rumen-protected amino acid supplementation to maintain health, metabolism, and production during periods of environmental heat stress and, furthermore, cope with the ongoing challenge of escalating global temperatures that the livestock industry is facing.

### 2.2. Fatty Acid Supplementation

Fatty acids are considered sources of energy; hence, their economic value derives largely from their ability to increase net energy of lactation (NEL) intake by dairy ruminants [119]. Among fatty acids, unsaturated fatty acids (UFA) are the main components of cell membranes. Their composition influences the function of cells and organs, along with their important role in physiological and metabolic processes, such as cellular membrane integrity, lipid metabolism, energy partitioning, hormonal pathways, oxidative stress and inflammatory pathways, and immune responses, blood coagulation and vascular resistance, enzyme activities, cell proliferation and differentiation, and receptor expression, thus regulating an organism's metabolic, immune, and antioxidant status [120,121,122]. Linoleic acid (LA, C18:2n-6) and a-linolenic acid (ALA, C18:3n-3), considered essential FAs (EFAs), are incorporated into membranes and are precursors for other important unsaturated fatty acids (of the n-6 and n-3 fatty acid families). EFAs are stored in tissues as triacylglycerols; however, their key roles are incorporation into membrane phospholipids and conversion to very long-chain fatty acids and eicosanoids, commonly known as prostaglandins and leukotrienes [123], which serve to both promote and inhibit inflammation [124].

#### 2.2.1. Fatty Acid Supplementation during Transition Period Inruminants

During the transition period in dairy cows, dietary supplementation of omega-3 and conjugated linoleic fatty acids (CLA) and inhibitors of lipolysis (i.e., fatty acid release), such as niacin, can effectively modulate immunometabolism function [125]. Polyunsaturated fatty acids (PUFAs) supplementation is regarded as an efficient strategy to mitigate the negative effects of negative energy balance and improve immune function in high-yielding periparturient dairy cows, due to its anti-inflammatory, immunomodulatory, and antioxidant properties [123,124,125]. Abomasal infusion of EFA (78 g/d linseed plus 4 g/d safflower oil) or conjugated linoleic acids (CLA; isomers cis-9,trans-11 and trans-10,cis-12 CLA; 38 g/d) or both EFA + CLA (120 g/d) in dairy cows from day 63 antepartum until day 63 postpartum had minor and time-dependent effects in the prevention of early lactation-induced oxidative stress, as indicated from markers of oxidative stress in plasma, erythrocytes, and liver [126]. However, the same authors found that EFA + CLA supplementation induced milk fat depression, increased energy balance, decreased postpartum NEFA and triglyceride concentration, reduced markers of inflammation (i.e., haptoglobin and paraoxonase), and attenuated hepatic lipid accumulation and ketosis in early lactation dairy cows [127]. According to the authors, CLA and EFA supplementation appears to be a strategic tool during the transition phase to conserve metabolisable energy and to reduce immune systems and metabolic incidences. Moreover, peripartum cows that were abomasally infused with trans-10, cis-12 CLA isomer (10.0 g/d) for 5 d presented decreased plasma NEFA concentrations and tended to have reduced (24%) lipolytic response after an epinephrine challenge [128]. Furthermore, supplementation of dairy cows during the transition period with ALA from flaxseed (80 and 220 g ALA/day per cow prepartum and postpartum, respectively) decreased the incidence of ketosis and severe metritis, reduced mortality, and tended to enhance fertility performance [129]. Intravenous infusion of emulsified fish oil rich in long-chained n3-PUFAs (EPA and DHA) at 12, 24, and 48 h after calving resulted in enhanced milk production and reduced inflammatory status and oxidative stress [130], although rumen protected, as Ca-salts, CLA supplementation (provided 30.4 g/d of CLA and the four predominant CLA isomers *trans*-8, *cis*-10 (9.2%), *cis*-9, *trans*-11 (25.1%), *trans*-10, *cis*-12 (28.9%), and *cis*-11, *trans*-13 (16.1%)) of dairy cows from 2 week prepartum to 20 week postpartum failed to affect postpartum concentrations of glucose, NEFA, and βHBA in plasma and hepatic content of glycogen and triglycerides [131].

Flaxseed ((omega-3)) dietary supplementation, as a rich source of n-3 FA, in the diet of dairy cows 6 weeks prepartum to 28 days of lactation (FL; 3.3 and 11.0% of the dry matter in prepartum and postpartum diets, respectively), was a useful strategy to increase liver concentrations of glycogen and decrease those of triglycerides after calving, which may help to prevent the development of fatty liver in the transition dairy cow [132]. Furthermore, crushed flaxseed supplementation (750 g/day/cow) of dairy cows 3 weeks pre- and 3 weeks postpartum improved cows’ metabolic status as indicated by the lower serum concentration of NEFA and βHBA and the increase in glucose concentration, although body weight and body condition score before and after parturition were similar between treatment groups, which, according to authors, reflects a better energy balance of supplemented cows relative to unsupplemented ones. Moreover, uterine involution was completed well within 30 days postpartum in all the cows in the flaxseed-fed group compared with the control group, and the size of dominant follicle and corpus luteum was also significantly higher in flaxseed-supplemented cows, which in turn resulted in higher concentrations of plasma progesterone [133].

In another study, supplementation of transition dairy cows (35 days prepartum to 70 days postpartum) with a medium chain saturated fatty acid mixture (20 g MCFA mixture every day/cow consisting of 10% octanoic acid, 7% decanoic acid, 17% lauric acid, and 66% carrier) improved the immune function of transition dairy cows, as indicated by the downregulation of the level of serum myeloperoxidase (MPO) and amyloid A (SAA) at early lactation, which are regarded as related immune markers [134].

In buffaloes, fat supplementation is at 100 g/d for 30 days before parturition and 15 g/kg milk yield per day for 120 days. Calf birth weight was higher in the bypass fat-supplemented group. Reproductive disorders like calf mortality, retention of placenta, metritis, and pyometra were also reduced to a great extent. Similarly, the days required for the appearance of first postpartum heat were reduced in the bypass fat-supplemented group. The length of the first and second estrous cycles was similar in both groups. The service period and number of artificial inseminations per conception were reduced in the bypass fat-supplemented group [135].

The effects of fatty acid supplementation on various physiological functions of ruminants during the transition period to cope with metabolic and the relative immune and antioxidant disturbances are summarized in Table 2.

In conclusion, fatty acid supplementation of dairy cows, in the form of PUFAs, during the transition period could be an effective dietary tool to support the immunometabolic function towards a successful transition to lactation.

#### 2.2.2. Fatty Acid Supplementation to Ameliorate the Effects of Heat Stress in Ruminants

The provision of dietary fatty acid supplementation to sustain the welfare and production performance of ruminants under high ambient temperatures has been suggested from various studies as a nutritional strategy. Polyunsaturated fatty acid supplementation has been investigated as a dietary tool to help dairy ruminants balance the negative effects of the heat stress on their physiological and immunological responses. Caroprese et al. (2009) [136] found that supplementation of dairy cows with n-3 PUFA as whole flaxseed (6.50 g/100 gr of dry matter of total ratio) enhanced immune responses of dairy cows exposed to high ambient temperatures, and it was considered an efficient strategy to increase both milk fat and protein content and milk yield and improve the FA profile and the CLA content of milk [137]. Furthermore, CLA supplementation, in the form of Ca-CLA, at doses of 200 or 400 g/day, had positive effects on heat-stressed dairy cows after 2 weeks of feeding since supplemented cows had higher plasma concentrations of K, Na, Ca, and Cl relative to non-supplemented cows, suggesting that CLA supplementation would moderate harmful changes in electrolytes during summer. Moreover, CLA supplementation reduced the concentration of plasma aspartate aminotransferase and creatine kinase and increased thyroxin concentration, thus alleviating body damage in hot weather. Overall, according to the authors, the addition of CLA moderated the negative responses in heat-stressed cows by regulating body temperature and affecting plasma enzymes, electrolytes, and hormones [139]. On the contrary, fish oil supplementation (1.10 g/100 gr of dry matter of total ratio) as a source of long-chain fatty acids enhanced the n-3 PUFA, EPA, and DHA contents of milk but did not affect the immune responses of cows under hot stress conditions [136,137].

In dairy sheep that were exposed to solar radiation and had mean THI above 80, whole flaxseed supplementation as a source of n-3 PUFA resulted in lower values of respiration rate and rectal temperature, a higher immune response, and higher cortisol, glucose, Na, and Cl concentrations. Thus, according to the authors, flaxseed supplementation during heat stress could be a strategy to sustain body thermal balance and humoral immune responses and enhance Na and Cl concentration in sheep under heat stress [139]. Furthermore, in another similar study, flaxseed supplementation as a source of PUFA helped sheep cope with heat stress, reduced sheep respiration rate, elicited activation of the hypothalamic pituitary adrenal axis by enhancing cortisol secretion, after adrenocorticotropic hormone (ACTH) challenge, probably to meet increased energy demand for thermoregulation, helped to sustain in vivo cell-mediated immune responses to phytohemagglutinin and humoral immune responses, as indicated by the reduced concentrations of Interleukin (IL)-13 that were connected to the recuction of heat shock protein (HSP) 70 and increased concentrations of IL-10, related to the increase in HSP 90 [140]. Whole flaxseed supplementation, as a source of PUFA, of lactating sheep exposed to high ambient temperature sustained animal welfare in terms of reduction of the respiration rate and of increased milk and fat yield. However, seaweed *Ascophyllum nodosum* supplementation, as a source rich in PUFA, EPA, and antioxidants, failed to sustain ewes’ welfare, as indicated by the increased respiration rate and rectal temperature and the lowest value of the body condition score [141].

In summary, fatty acid supplementation is regarded as an intervening feeding practice to ensure body thermoregulation and enhance the immunometabolism and antioxidant activity of heat-stress ruminants, thus ensuring animals’ comfort and welfare, as summarized in Table 2.

### 2.3. Feed Additive Supplementation

#### 2.3.1. Feed Additive Supplementation during Transition Period in Ruminants

The use of feed additives in transition ruminant feeding to alleviate stress has been considered the gold standard. Minimizing metabolic disturbances and alleviating ruminants in transition from any stress related to the adaptation of ruminal flora to a modified ration and an escalating milk yield is a strategy that is guaranteed to safeguard the health, productivity, and welfare of the animals. Silvestre et al. (2023) [142] and Humer et al. (2018) [143] both emphasize the importance of rumen health and the role of feed additives, such as ionophores, probiotics, and plant extracts with antimicrobial effects, in mitigating subacute ruminal acidosis by aiding in the gradual adaptation of microbiota and epithelium in the rumen. Nikanova et al. (2020) [144] highlight the potential of energy feed additives and high protein feed concentrate in improving productivity and metabolic homeostasis in dairy cattle by boosting appetite after parturition, stabilizing oxidative stress, and low immunity. Such immunomodulatory feed additives have been shown to regulate metabolic and immunological responses to sub-acute ruminal acidosis in lactating dairy cows, who, as a result, often suffer from subsequent systemic inflammation during transition [145]. Apart from nutritional stressors, those of environmental origin exhibit significant pressure on the homeostasis of ruminants in transition, while the use of yeast, niacin, zinc, and chromium, among others, has been shown to reduce heat stress in heifers [146].

##### Minerals

Mineral metabolism alters significantly during the transition period in ruminants in an effort to support milk production and, at the same time, circumvent any impairment of metabolic processes [21]. In small ruminants, mineral supplementation is recommended during any transition events that may cause stress to the animals, with specific minerals such as sodium (Na), phosphorus (P), copper (Cu), cobalt (Co), zinc (Zn), selenium (Se), and iodine (I) being crucial [147]. Trace mineral activity is critical for all ruminants, as they play significant roles in gluconeogenesis (involving Co-containing methylmalonyl-CoA mutase), ureagenesis (facilitated by manganese (Mn)-containing arginase), synthesis of vitamin B12, and hemoglobin production, and additionally, they are vital for the activity of enzymes like superoxide dismutases (Zn and Cu), catalases (ferric), and certain transcription regulators (Zn) [148]. Micronutrient deficiencies, particularly in magnesium (Mg), selenium (Se), copper (Cu), chromium (Cr), and zinc (Zn), lead to oxidative stress and immunosuppression in transition cows, initiating as a subclinical status and then progressing to clinical manifestation, as in the case of hypocalcemia and ketosis [149]. Taking into account that the majority of micronutrients form the active centers of antioxidant enzymes, it is easy to deduce that their supplementation can suppress the effects of ROS [149]. Organic trace element supplements, in particular selenium, copper, chromium, and zinc, have been found to improve udder health, lameness, and reproductive performance in transitioning dairy cows [150]. The supplementation of dairy cows’ rations with organic zinc has been found to dimidiate new intramammary infections, while the inclusion of Cu, Zn, and Se daily did reduce Somatic Cell Count (SCC) in milk significantly [150]. As a mineral that holds a significant part in the keratinization process during hoof formation, Zn, when added to dairy cows’ nutrition, is connected to lower lameness events, digital dermititis, foot rot, laminitis, and related claw disorders [150]. Regarding reproductive performance, the key management strategy on mineral use appears to be the replacement of inorganic sources with organic ones, which seem to contribute to improving compliance rates and reducing days to first service, although statistically significant results are yet to be produced from such trials [135,150]. The roles of calcium, magnesium, phosphorus, and dietary anion cation difference in influencing the pathophysiology and incidence of hypocalcemia in transition cows have also been highlighted [151], and furthermore, an optimal supplementation of complexed trace minerals has been shown to improve immune function and reduce the risk of uterine infections in dairy cows by limiting hepatic cell damage while enhancing liver function and restricting the degree of inflamation associated with the metabolic processes put in practice during transition and reinforcing animals’ anti-inflammatory response [148]. Lastly, micronutrients with antioxidant activity, including Cu, Zn, Se, and vitamins A, C, and E, have been found to improve dry matter intake, nutrient digestibility, and reproductive performance indices (especially resumption of oestrus) in ruminants under stress, acting as antioxidant agents, e.g., selenium and vitamin E, and protecting from oxidative stress and lipid peroxidation complementarily, also protecting oocyte maturation and embryo development, while modifying the metabolic activities of the gut microbiota and improving the intestinal barrier [152].

##### Probiotics

A range of studies have highlighted the potential of nutritional interventions through the employment of probiotics in managing stress in ruminants, as their effect on energy intake is crucial and, when administered in a proper way, as part of a balanced ration, according to the needs of the animal at a specific production stage, yield, age, and health status, can regulate the digestive tract’s microbial balance and enhance stress regulation. Nutritional supplementation with probiotic mixtures (*Enterococcus faecium*, *Bifidobacterium bifidum*, *Lactobacillus acidophilus*, *Streptococcus thermophilus*, *L. rhamnosus*, *L. bulgaricus*, and *L. plantarum*) has been shown to address metabolic stress and promote effective nutrient utilization in dairy cattle in transition, while increasing dry matter intake and milk yield [153]. Similarly, a commercial formulation with probiotics (*Saccharomyces boulardii*, *Lactobacillus acidophilus*, and live yeast culture of *Saccharomyces cerevisiae*) has been found to alleviate walking stress in Hassani goats [154]. The use of probiotics in small ruminant nutrition has also been linked to improved health, productivity, and immunity, and most importantly in the case of transition ewes and does, with a stabilization of rumen pH, leading to a more regulated rumen function and lowering the risk of sub-acute ruminal acidosis. Specifically, goats consuming diets supplemented with yeast cultures have been found to exhibit higher milk fat content as well as a limited milk production increase [155]. Furthermore, the effectiveness of a probiotic based on lactic acid bacteria in stabilizing ruminal processes in dairy cows during diet changes has been demonstrated when, after adding a probiotic consisting of lactobacilli and bifidobacteria (total concentration of 5–10^9^ CFU) during a change in the ration, such as introducing a new batch of corn silage, dry matter intake occurred sooner than in animals not receiving probiotics, and by the fifth day after the ration change, those cows consumed the same quantity as pre-change, while rumination frequency and feces consistency remained normal [156]. Nutraceuticals, including probiotics, have been suggested as a means of modulating the immune response and metabolic activity altered by inflammatory responses in dairy cows during the transition period by implementing their antioxidant effects [22].

##### Prebiotics

The potential of prebiotic supplementation in managing metabolic stress and immune dysfunction in transition cows has often been highlighted [22,157]. Similarly, prebiotic supplementation has been found to enhance antioxidant status and reduce inflammation in transition dairy goats [158]. The use of prebiotics in goats has also been shown to improve ruminal microflora and enhance digestion [159]. Similarly, prebiotics have been shown to modulate the immune response and metabolism in dairy cows during the transition period, potentially by enhancing humoral and mucosal immunity in combination with reducing inflammation by increasing feed intake at the onset of lactation [22]. These findings suggest that prebiotic supplementation can be an effective strategy for alleviating stress in ruminants during transition. For example, beta-1,3-glucan, a dietary fiber polysaccharide, supplementation has been found to improve productivity, immunity, and antioxidative status in periparturient dairy cows, while also elevating colostrum quality, by regulating metabolism disorders caused by negative energy balance, such as oxidative stress due to the mobilization of body fat, thus increasing milk production while reducing SCC and elevating immunoglobulin concentration in colostrum [157]. When berberine, a natural isoquinoline alkaloid, is added to transition dairy goats’ diet, it has been found to mitigate oxidative stress and inflammation while improving energy balance, as derived by the increase in de novo and preformed fatty acid (FA) concentrations (sum of FA > C16) [158,160]. Furthermore, the use of prebiotics and probiotics has been shown to alleviate production losses associated with mycotoxicosis in goats [161]. Lastly, a combination of both prebiotic (mannan and B glucan) and probiotic (lactobacillus acidophilus with Bacillus subtilis and Bacillus licheniformi) treatments has been found to improve ruminal microflora and enhance digestion in goats suffering from simple indigestion [159].

##### Essential Oils/Herbs

Uyarlar et al. (2024) [162] have suggested that essential oil supplementation, in the form of a blend of peppermint, eugenol, anise, and thyme essential oils at a dose of 3 g/day per animal in the total mixed ration (TMR), improves immune status and energy metabolism in dairy cows during the transition period, with no adverse effects on suckling calves. Similarly, Braun et al. (2018) [163] reported that essential oil supplementation (incorporation of a blend of EOs containing eugenol, menthol, and anethol) in dairy cows enhanced milk yield, fat and protein content, and feed efficiency. In small ruminants, Caroprese et al. (2023) [164] highlighted the potential of essential oils (such as oregano, thyme, eucalyptus, and garlic) in modulating rumen fermentation and improving animal health and welfare. However, Tassoul and Shaver (2009) [165] found that essential oil supplementation (in the form of a blend of natural and natural-identical EO compounds that included thymol, eugenol, vanillin, and limonene) reduced dry matter intake in early lactation dairy cows, still without affecting milk yield. Despite these mixed findings, Lopreiato et al. (2020) [22] and Dorantes-Iturbide et al. (2022) [166] both concluded that essential oil supplementation improved animal performance in terms of weight gain and milk yield, meat and milk quality, and antioxidant status, as well as in rumen parameters and serum metabolites in small ruminants.

##### Phytobiotics

Phytobiotics, considered a type of nutraceuticals, are plant-derived natural bioactive components that can be used as feed additives in farm animals’ rations and have the ability to improve their health and productivity. Phytochemical supplementation can effectively alleviate stress in transition cows, sheep, and goats, as these supplements, including polyunsaturated fatty acids, vitamins, dietary amino acids, and phytochemicals, have been found to modulate energy homeostasis and address metabolic issues in transition ruminants. Phytobiotics have been shown to have host-mediated effects in ruminants, including immune regulation and energy partitioning for milk production [167]. Specific phytobiotic products, such as poly-herbal formulations (Boswellia serrata and Berginia ciliata), have been shown to reduce stress and boost the immunity of dairy cows during the transition period, even providing an improvement of udder health status [168]. A phytobiotics-rich herbal mixture of rosemary, cinnamon bark, turmeric, and clove bud has been found to improve performance and udder health by aiding in somatic cell count reduction, especially in animals with high SCC, and metabolic status in dairy cows, effects that could be attributed to the high quantity of phenolic compounds in the mixture, which in turn provided for potent lipophilic and hydrophylic antioxidant activity [169]. Ding et al. 2022 [170] suggested that the supplementation with rutin, a flavonoid of transition sheep diets, had positive effects against inflammation, oxidative stress status, and anti-apoptotic activity in the mammary gland. The use of phytobiotics in ruminant nutrition has been particularly effective in reducing cortisol concentration, modulating energy homeostasis, improving immune status, increasing feed intake and milk production, and modifying rumen flora, on top of their antimicrobial activity [22,167,168,169].

##### Enzymes

The exogenous enzymes supplemented in ruminants’ diets include amylolytic enzymes [such as α-amylase (EC 3.2.1.1)], proteolytic enzymes [such as serine endopeptidase of the subtilisin family (EC 3.4.21.62)], β-glucanase, xylanase [endo--1,4-xylanase (1.43.2.1.8), -D xylan xylanohydrolase, EC-D-xylosidase (1,4--xylan xylanohydrolase, EC 3.2.1.37)], and β-mannanase, which have been found to improve feed intake, nutrient digestibility, and growth performance [171]. These enzymes are usually extracted from specific bacterial and fungal species, as well as some yeasts [171]. Similarly, sheep and goats fed with roughages treated with exogenous enzymes prior to consumption have exhibited improved nutrient digestibility and growth performance [172]. Lastly, the addition of fibrolytic and amylolytic enzymes in sheep diets has been found to improve nutrient intake and digestibility, nitrogen balance, and ingestive behavior, which in turn enhances rumen function [173].

To conclude, feed additives might draw attention as feeding supplements during the transition period of ruminants due to their potential health benefits and metabolic responses, reflecting an increase in productive and reproductive performances and overall wellbeing. The effects of their supply on transitional ruminats are summarized in Table 3.

#### 2.3.2. Feed Additives Supplementation during Heat Stress in Ruminants

##### Minerals

Son et al., 2022 [174] found that high concentrations of se, Cu, and Zn reduced oxidative stress and metabolic alterations, which are closely associated with heat stress, in Holstein and Jersey steers by increasing serum superoxide dismutase, an antioxidant enzyme, concentrations while decreasing oxidative stress-induced heat shock protein concentrations. Similarly, Conte et al. (2018) [50] and Pawar et al. (2018) [52] both highlighted the importance of mineral feeding in mitigating the negative effects of heat stress on ruminants, especially Se, due to its support of the organism’s antioxidant defense by acting as a component of glutathione peroxidase, which exterminates free radicals, improving mammary gland immunity, and lowering lipid peroxidation. Selenium supplementation appears to offer an improvement of physiological thermoregulation, which, in turn, enhances milk composition [79]. It is also important to emphasize that mineral supplemantation offers buffering advantages that are crucial for effective rumen function under heat stress conditions [175]. Additionally, the supplementation of heat-stressed dairy cows with NaHCO3, K2CO3, and KHCO3 as well as Cr appears to improve feed intake, milk yield, and insulin sensitivity [175]. Furthermore, mineral and antioxidant supplementation, through a bundle of zinc, cobalt, chromium, selenium, and vitamin E, allowed for the body condition score of heat-stressed animals to remain consistent, as was estrogen detection and adaptive capability (evaluated through hormonal responses) in Malpura ewes [176]. Sunilkumar et al. (2011) [177] demonstrated the potential of dietary supplementation in moderating serum electrolyte levels and boosting cell-mediated immunity in buffaloes. Lastly, the introduction of a thermoregulatory mineral supplement containing calcium, chlorine, sodium, and potassium in the ration of dairy cows under a warm climate reduced their rectal temperature (by 1 degree Celcius) in comparison to the controls, although all animals remained within the normal range [178].

##### Probiotics

Probiotic supplementation has been shown to alleviate heat stress in ruminants, improving thermocardiorespiratory responses and hemobiochemical parameters in goats [179]. Probiotics, in the form of *Bacilus subtilis* and propionic bacteria, also enhance productive performance, digestibility, and physiological responses by having a positive effect on blood metabolites in beef bulls [180]. Specific probiotics, such as *Saccharomyces cerevisiae* and *Clostridium butyricum*, have been found to improve rumen fermentation by significantly increasing rumen pH and total volatile fatty acids, as well as cellulolytic enzyme activity and growth performance, by improving dry matter intake and average daily gain in heat-stressed goats [181,182,183]. A combination of live yeast and yeast cell wall products has been shown to mitigate the negative impacts of heat stress in feedlot heifers by lowering cortisol concentration and drinking a larger volume of water in more drinking bouts, which was also connected to a decrease in vaginal temperature [184]. Similarly, Shaker et al. (2008) [179] and Abdel-Fattah et al. (2008) [154] found that probiotics can reduce the burden of heat stress in goats. Natural additives like propolis extract have also been suggested as potential solutions to mitigate the effects of heat stress in ruminants, as they often have antibacterial and rumen regulatory action due to their ionophoric properties [185]. However, the beneficial effects of probiotics can vary depending on factors such as microbial strain, dosage, diet, supplementation protocol, and individual animal characteristics [186].

##### Prebiotics

Prebiotic supplementation has been shown to have a positive impact on ruminants under heat stress, an effect that is fortified when prebiotics are combined with probiotics (synbiotics) [187]. Studies on beef bulls [180] have demonstrated improvements in growth performance, nutrient digestibility, and physiological responses. These benefits are attributed to the positive effects of prebiotics on the intestinal microbiota, gut morphology, and oxidative status. Specific prebiotics, such as mannan oligosaccharides, fructooligosaccharides, and galactooligosaccharides, have been found to stimulate the growth of beneficial bacteria and inhibit pathogenic microbiota in the gastrointestinal tract of ruminants [188]. In lambs, prebiotics (mannan oligosaccharides in particular) appear to enhance dry matter intake and subsequently average daily gain [187].

##### Essential Oils/Herbs

Caroprese et al. (2023) [164] and Dorantes-Iturbide et al. (2022) [166] both found that essential oil supplementation (thyme, fennel, ginger, black seed, and Eucalyptus oil) can optimize rumen fermentation, gut health, and productivity, including weight gain, milk production, and meat quality in both cows and small ruminants. Additionally, essential oils have the ability to modify the microbial populations of the intestinal tract, enhance barrier integrity, and, in this way, improve performance in sheep and goats [164]. Reza-Yazdi et al. (2014) [189] and Andri et al. (2020) [190] further demonstrated that essential oils such as bundles of cinnamaldehyde, eugenol, peppermint, coriander, cumin, and lemongrass can mitigate the negative effects of heat stress on feed consumption and milk production in dairy cows by modifying the proportions of volatile fatty acids in the rumen and improve growth performance in small ruminants. Chávez Soto et al. (2021) [191] emphasized the need for further research to optimize the use of essential oils in small ruminant feeding. However, the evidence on the growth-promoting effects of essential oils in small ruminants is inconclusive, with some studies reporting no significant impact on dry matter intake and feed conversion ratio [190].

##### Phytobiotics

Phytobiotics, including plant-derived bioactive compounds, have been shown to have a range of beneficial effects in ruminants, including antimicrobial activity, modulation of rumen fermentation, and regulation of immune responses, all of which are of vital importance for heat-stressed animals [167]. These effects can help alleviate the negative impacts of heat stress, such as inflammation and oxidation in dairy heifers, that due to body surface are more severely impacted by heat stress, through the use of a mixture containing garlic oil, anise oil, cinnamaldehyde, rosemary, and thyme blend [192]. Bąkowski and Kiczorowska (2021) [193] highlight the potential of these compounds to improve animal health and production efficiency, specifically noting their ability to increase feed intake and prevent irritation and diarrhea, which are very often profound impacts of heat stress. Specific phytobiotics, such as herbal mixtures containing cinnamon, turmeric, rosemary, and clove buds, have been found to improve growth performance and antioxidant status in heat-stressed lambs [194]. Similarly, phytobiotics, which exhibit probiotic activity, have been shown to improve growth performance, nutrient digestibility, and physiological responses in beef bulls under heat stress conditions [180]. The use of phytobiotics, including propolis extract, as a heat stress attenuate agent in ruminants has also been explored [185]. In dairy goats under warm conditions, but not heat stress, daily supplementation of their TMR with a mixture of organic acids (sorbic and citric acid), thymol, and vanillin increased milk fat content and coagulation index [195].

##### Enzymes

Gado et al. (2014) [196] found that ration supplementation with exogenous enzymes from anaerobic bacteria increased plasma parameters, boosting glucose and milk production and enhancing protein and lactose concentration in dairy ewes during summer and under heat stress conditions. Sujani and Seresinhe (2015) [171] and Meale et al. (2014) [197] both highlighted the potential of exogenous enzymes to improve feed intake, nutrient digestibility, and growth performance in ruminants due to their interaction with rumen microbiota, although usage efficacy must be taken into consideration as not all enzymes exhibit increased activity in all categories of ruminants (especially in sheep and goats). Finally, Shakya et al. (2019) [198] emphasized the potential of enzyme supplementation, particularly fibrolytic, proteolytic, and amylolytic enzymes, to enhance livestock production, as they can assist in increasing milk fat content in the milk of heat-stressed dairy cows, where fat depression is one of the most important downsides in terms of productivity. In this case, fibrolytic enzymes that can modify the ratio of propionate to acetate volatile fatty acids in the rumen increase milk fat concentration.

Consequently, various nutraceuticals might be, among others, a potential nutritional tool to alleviate heat stress symptoms of ruminants and efficiently affect animals’ thermoregulation, metabolism, physiology, health, and production, as summarized in Table 3.

**Table 3:** Summary of the physiological and performance responses after dietary supplementation of ruminants with feed additives at transition period and under heat stress.

Feed Additives	Observed Effects	Refs.
During transition period		
Minerals		
Na, P, Cu, Co, Zn, Se, I, Mn, Cr, Ca	Improvement of liver function, decrease in inflammation, and oxidative stress. Alleviation of the pathophysiology and incidence of hypocalcemia in transition cows. Improvement of dry matter intake, nutrient digestibility, and reproductive performance indices.	[147,148,149,150,151,152]
Probiotics		
*E. faecium*, *B. bifidum*, *L. acidophilus*, *S. thermophilus*, *L. rhamnosus*, *L. bulgaricus*, *L. plantarum*, Yeast	Promotion of effective nutrient utilization, increase in dry matter intake, milk yield, milk fat content, alleviation of metabolic stress in transitional dairy cattle. Normal rumination frequency and feces consistency.	[153,154,155,156]
Prebiotics		
Berberine, mannan, B glucan	Enhancement of antioxidant status, reduction of inflammation, improvement of ruminal microflora digestion, alleviation of morbidity, and production losses due to *Haemorrhagic Enteritis* in transition dairy goats.	[158,159,160]
Various mixes	Imrovement of the immune response and metabolism in transitional dairy cows.	[22]
Beta-1, 3-glucan	Improvement of productivity, immunity, antioxidant status, and colostrum quality in periparturient dairy cows.	[157]
Essential oils/Herbs		
EOs (eugenol, menthol, anethol) EOs (oregano, thyme, eucalyptus and garlic) EOs (thymol, eugenol, vanillin, limonene)	Enhancement of milk yield, fat and protein content, and feed efficiency in dairy cows.	[163]
	Modulation of rumen fermentation and improvement of animal health and welfare.	[164]
	Reduction of dry matter intake; no effects on milk yield in early lactation dairy cows.	[165]
	Improvement of antioxidant status, rumen parameters, serum metabolites, and weight gain, milk yield, meat and milk quality in small ruminants.	[22,166]
Phytobiotics		
Poly-herbal formulations (Boswellia and Berginia ciliata)	Reduction of stress, boost of immunity improvement of udder health status of dairy cows during the transition period.	[168]
Herbal mixture of rosemary, cinnamon bark, turmeric, clove	Improvement of metabolic status, udder health, and performance in dairy cows.	[169]
Rutin	Positive effects against inflammation, oxidative stress status, and anti-apoptotic activity in the mammary gland of transition sheep	[170]
Enzymes		
Amylolytic, proteolytic β-glucanase, xylanase, and β-mannanase, exogenous enzymes	Improvement of feed intake, nutrient digestibility, and growth performance in sheep.	[171,172]
fibrolytic and amylolytic enzymes	Improvement of nutrient intake and digestibility, nitrogen balance, and ingestive behavior; enhancement of rumen function in sheep.	[173]
During heat stress		
Minerals		
Se, Cu, zinc	Reduction of oxidative stress, increase in serum superoxide dismutase, decrease in heat shock protein, metabolic alterations associated with heat stress steers.	[174]
Se	Increase in glutathione peroxidase, decrease in free radicals, decrease in lipid peroxidation, improvement of mammary gland and immunity, improvement of thermoregulation, and milk yeild.	[50,52]
NaHCO3, K2Co3, KHCO3	Improvement of feed intake, milk yield, and insulin sensitivity.	[50]
Zn, Co, Cr, Se	Improvement of antioxidant status and maintenance of body condition score of heat-stressed ewes.	[176]
Probiotics		
*B. subtilis*, propionic bacteria	Enhancement of blood metabolites, physiological responses, productive performance, and digestibility in beef bulls.	[180]
*S. cerevisiae* and *C. butyricum*	Improvement of rumen fermentation, increase in rumen pH and total volatile fatty acids, cellulolytic enzyme activity, and growth performance in heat-stressed goats.	[181,182,183]
Live yeast and yeast cell wall product	Decrease in cortisol concentration and vaginal temperature, increase in drinking water volume in feedlot heifers.	[184]
Various probiotics	Mitigation of heat stress effects in goats.	[154,179,185,186]
Prebiotics		
Mannan oligosaccharides, fructooligosaccharides, galactooligosaccharides	Positive effects on the intestinal microbiota, gut morphology, and oxidative status, improvement in growth performance, nutrient digestibility, and physiological responses. Stimulation of the growth of beneficial bacteria and inhibition of pathogenic microbiota in the gastrointestinal tract, improvement of performance.	[187]
Essential oils/Herbs		
Thyme, fennel, ginger, black seed, Eucalyptus oil	Modification of microbial populations of the intestinal tract, enhancement of barrier integrity, optimization of rumen fermentation, gut health and productivity, weight gain, milk production, and meat quality in both cows and small ruminants	[164,165]
Cinnamaldehyde, eugenol, peppermint, coriander, cumin, lemongrass	Mitigation of the negative effects of heat stress on feed consumption and milk production in dairy cows. Modification of the proportions of volatile fatty acids in the rumen and improvement of growth performance in small ruminants.	[189,190]
Phytobiotics		
Mixture (garlic oil, anise oil, cinnamaldehyde, rosemary, and thyme blend)	Alleviation of the inflammation and oxidation of heat-stressed animals, prevention of irritation and diarrhea, improvement of animal health and production efficiency.	[192,193]
Cinnamon, turmeric, rosemary, and clove buds	Improvement of growth performance and antioxidant status in heat-stressed lambs.	[194]
Phytobiotics which exhibit probiotic activity	Improvement of growth performance, nutrient digestibility, and physiological responses in beef bulls under heat stress conditions.	[180]
Enzymes		
Exogenous enzymes from anaerobic bacteria	Increase in plasma parameters, boosting glucose and milk production, enhancing protein and lactose concentration in heat-stressed dairy ewes.	[196]
Exogenous enzymes	Improvement of feed intake, nutrient digestibility, and growth performance in ruminants due to their interaction with rumen microbiota in heat-stressed ruminants.	[171,197]
Fibrolytic, proteolytic, and amylolytic enzymes	Modification of the ratio of propionate to acetate volatile fatty acids in the rumen, increase in milk fat concentration, and production of heat stressed dairy cows.	[198]

## 3. Breeding Strategies as a Tool to Maximize Effects of Dietary Interventions

The implementation of specific breeding strategies in ruminant production systems can be a useful tool to increase animal resilience, alleviate stress, and improve welfare. One approach to improving welfare is the selection of breeds or selection within breeds for lower levels of basic metabolism. Animals with lower metabolic rates are often more resilient to fluctuating environmental conditions, such as extreme temperatures or low feed availability [46], and can also lead to improved feed efficiency, as less energy is spent on basic metabolic processes, thus reducing feed costs and the environmental impact associated with feed production. Additionally, improved feed efficiency can lead to better animal health and welfare for animals, as they are better able to meet their nutritional needs without excessive energy expenditure [199,200].

Other examples of traits that can be incorporated in a breeding program are calm temperament, which has been shown to reduce stress responses and improve handling efficiency in cattle [201], reduced stress responses [202], and improved coat composition and structure in goats which can enhance their ability to withstand harsh environmental conditions [203].

### Utilization of Local Breeds

Local ruminant breeds have over time become highly adapted to their native environment and are therefore particularly well suited to production systems with low inputs, limited resources, harsh climates, and limited feed availability. By using only local breeds or integrating them into breeding programs, producers can benefit from their adaptability and resilience and reduce the need for external inputs such as medicines and supplementary feed. In areas with challenging climates, such as arid or mountainous regions, local ruminant breeds often have better adaptive traits compared with exotic breeds. Small ruminants, for example, show a wide range of adaptive responses to overcome heat stress in tropical regions, initiating thermoregulatory responses to dissipate the extra heat as well as behavioral and zhysiological/biochemical changes [195]. Some examples of breeds tolerant to heat are Dorper, Meatmaster, and Namaqua. Another example is the Red Maasai and Texel sheep breeds that show increased resistance to gastro-intestinal nematodes [204]. Their ability to efficiently convert low-quality feed into products of high quality makes them valuable assets in sustainable agriculture. In addition, local breeds tend to be more resistant to disease and heat, reducing the need for labor-intensive management practices and veterinary treatments. Supporting the conservation and use of local breeds not only promotes efforts to preserve cultural heritage and biodiversity but also provides practical solutions to improve resilience and sustainability in ruminant production systems [205].

Although the selection of breeds with lower metabolic requirements shows promise for improving ruminant welfare, proper consideration of genetic selection and breeding strategies is required to maximize welfare benefits while minimizing potential disadvantages at the expense of other important traits. Furthermore, it is crucial to maintain genetic diversity within breed populations to ensure their sustainability and resilience as a measure against emerging threats such as climate change.

## 4. Conclusions and Perspectives

Dairy ruminants are vulnerable to various physical stress factors during their productive lifespan, such as weaning, parturition, early lactation, during transportation, etc. Additionally, intensification of production subjects them to high nutritional demands, along with high metabolic and physiological processes, whereas climate change and the associated ambient temperature escalation vulnerable animals to uncomfortable conditions under which various physiological, immunometabolic, and antioxidant processes are activated to cope with the increasing body temperature. Among other practices, nutrition is regarded as an efficient tool to alleviate ruminants from stress factors. As concluded in this manuscript, amino acids, fatty acids, high-PUFA feed resources, and feed additives such as minerals, pre- and probiotics, herbs and essential oils, phytobiotics, and enzymes are proposed as necessary feed regimes in ruminants’ diets to combat stress situations. These nutritional interventions have been proven from the studies to act at a cellular and molecular level; they act as activators of cell signaling that influence key metabolic pathways and important physiological functions; they modulate the metabolic, immune, and antioxidant system responses to alleviate the incidence of inflammation, immune dysregulation, and metabolic adaptation impairment under stress conditions. However, more research is needed to fully understand their exact regulatory mechanisms due to their difference in their origin and form, their complexity, and the difference in their functionality. Moreover, further experimental and in-field studies are needed considering the variabilityin the environmental conditions, the rearing systems, and the applied management practices, as well as the level of animal production. Afterwards, it is proposed the research and the application of the combination of these sources in ruminants’ diets to take advantage of their different functional properties on metabolism, immune, and antioxidant systems, so as animals’ cope with stress factors and be able to acquire optimum performance and health, and thus welfare, in the frame of the modern intensified production systems.

## Figures and Tables

**Table 1:** Summary of the physiological responses after dietary supplementation of ruminants with rumen-protected amino acids at transition period and under heat stress.

Amino Acid	Observed Effects	Refs.
Amino acid supplementation during transition period.		
RPM	Enhancement of immunometabolic status, reduction in liver proinflammatory signaling, increase in albumin concentration, neutrophil phagocytosis capacity, proliferative ability of peripheral blood T lymphocytes, and alleviation of hyper response to inflammatory cytokines.	[77,78,79,80,81,82,83]
RPM + RPC	Enhancement of immune function, elevation of IL-2, CD4+/CD8+ T lymphocyte ratio, and decrease in the (TNF-α) and IL-6 concentration.	[82]
RPM + RPL	Decrease in milk somatic cell count and improvement of immunity and health status.	[84]
RPM	Improvement of blood antioxidant status, increase in plasma ferric-reducing antioxidant power, β-carotene, tocopherol, total and reduced GSH, increasing total antioxidant capacity, GPX activity, vitamin E, and decrease in plasma reactive oxygen metabolites.	[78,80,81]
RPM	Positive effects on mammary glad antioxidant status and greater mRNA abundance of specific key target genes that regulate tissue’s antioxidant mechanisms.	[81]
RPM	Increase in liver function biomarkers, reduction in inflammation, and oxidative stress.	[77,79,80]
RPM, RPM + RPC	Alleviation of oxidative stress and enhancement of plasma glutathione transferase activity of periparturient ewes.	[85]
RPM, RPM + RPC	Alleviation of oxidative stress and increase in glutathione activity.	[86]
RPC	Improvement of liver biomarkers and metabolic stress and decrease in NEFA and βHBA levels.	[87,88,89,90,91,92,93,94,95,96,97]
RPM	Ιmproved follicular structures and increased production and reproduction outcomes in Murrah buffaloes.	[98]
RPM, RPM + RPC	Increased in GSH, SOD, TAS, decreased LPO, TNF- α, and haptaglobin. Increase in antioxidant status and immune response.	[99]
RPL, RPM, RPM + RPL	Improved DMI, milk yield and milk production efficiency, energy-corrected milk, and milk chemical composition. Greater peak milk yield, earlier milk peak, and higher fertility efficiency.	[100,101]
Gln	Increase in cytokine production by immune system cells in peripartum dairy cows.	[103]
RPGln	Enhancement of liver function, lower aspartate aminotransferase concentration, amelioration of metabolic stress, lower βHBA, and free FAs concentrations.	[104]
RPGln	Improved antioxidant status of the animals as indicated by the higher total antioxidant capacity (TAC) and GSH activity.	[105]
Amino acid supplementation to alleviate heat stress in ruminants.		
Lys, Met, Hist	Thermoregulation, decreased rectal temperature, and enhancement of intermediary metabolism in heat-stressed dairy cows.	[106,107]
Met, Arg (In vitro)	Improvement cell functions of heat-stressed bovine mammary epithelial cells and mammary metabolism.	[108]
RPM	Maintenance of mTOR homeostasis, insulin signaling, 1-carbon metabolism, and whole-blood antioxidant response of heat-stressed dairy cows.	[109]
RPM	Decreased mammary cell apoptosis to proliferation ratio, no significant effects on blood immune biomarkers of heat stress-challenged cows	[110]
RPM	Enhancement of plasma IgG concentration, increased milk protein content, and decreased plasma urea–nitrogen concentration in heat-stressed dairy goats.	[111]
RPGln	Enhancement of immune function and improvement of cell-mediated immune response of heat-stressed dairy cows.	[112]
Betaine	Anti-apoptotic properties, increase in cell proliferation, and preservation of gut tissue integrity of dairy cows.	[113]
Betaine	Thermoregulation, decrease in rectal and skin temperatures, decrease in respiration rates, and plasma NEFA concentrations of heat-stressed sheep.	[114]
Betaine	Improvement of milk yield and quality of heat-stressed grazing dairy cows.	[115]
Betaine	Increase in feed intake, rumen fermentation, apparent digestibility, improvement of the antioxidant profile, and serum metabolites of heat-stressed cows.	[116]
Betaine	Improvement milk fat. Lower rectal temperature, respiration rate, and pulse rate in heat-stressed buffaloes.	[117]

**Table 2:** Summary of the physiological responses after dietary supplementation of ruminants with fatty acids at transition period and under heat stress.

Amino Acid	Observed Effects	Refs.
Fatty acid supplementation during transition period.		
EFA, CLA, EFA + CLA	Minor prevention of oxidative stress, enhancement of markers of oxidative stress in plasma, erythrocytes, and liver.	[126]
EFA + CLA	Milk fat depression, increased energy balance, decreased postpartum plasma NEFA and triglyceride concentration, reduced markers of inflammation, attenuation of hepatic lipid accumulation, and ketosis in early lactation dairy cows.	[127,128]
ALA	Decrease in ketosis incidence and severe metritis, reduced mortality, and enhancement of fertility performance.	[129]
Fish oil (EPA and DHA)	Enhancement of milk production and amelioration of inflammatory and antioxidant status.	[130]
RPCLA (Ca-salts)	No effects of glucose, NEFA, and βHBA in plasma and hepatic content of glycogen and triglycerides concentration of dairy cows, peripartum.	[131]
Flaxseed	Increase in liver glycogen concentrations and decrease in triglycerides postpartum.	[132]
Crushed flaxseed	Improvement cows’ metabolic status peripartum, decrease in serum NEFA and βHBA concentration, and increase in glucose concentration.	[133]
MCSFA	Improvement of immune function and downregulation of serum myeloperoxidase level amyloid A of transition dairy cows.	[134]
Bypass fat	Higher calf birth weight, reduced calf mortality, retention of placenta, metritis, pyometra, and the service period and number of artificial inseminations per conception in transitional buffaloes.	[135]
Fatty acid supplementation to alleviate heat stress in ruminants.		
Whole flaxseed	Enhancement of immune responses of dairy cows, increase in both milk fat and protein content and milk yield, improvement of milk FA profile and CLA content.	[136,137]
Ca-CLA	Increase in plasma concentrations of K, Na, Ca, and Cl; decrease in plasma aspartate aminotransferase and creatine kinase; and increase in thyroxin concentration of heat-stressed cows.	[138]
Fish oil	Enhancement of n-3 PUFA, EPA, and DHA contents of milk; no effects on immune responses of heat-stressed cows.	[136,137]
Whole flaxseed	Reduction of the respiration rate and rectal temperatures; increase in cortisol after ACTH challenge, glucose, Na, and Cl concentration; and enhancement of cell-mediated immune responses to phytohemagglutinin and humoral immune responses in sheep.	[139,140,141]

## Data Availability

No new data were created or analyzed in this study.
